# Dr. Whitelaw Ainslie on the Materia Indica, &c.

**Published:** 1827-04

**Authors:** 


*Materia Indica; or, some Account of those Articles which are employed by the Hindoos, and other Eastern Nations, in their Medicine, Arts, and Agriculture; comprizing also Formulæ, with Practical Observations, Names of Diseases in various Eastern Languages, and a copious List of Oriental Books immediately connected with General Science, &c. &c.* By Whitelaw Ainslie, M.D. M.R.A.S. late of the Medical Staff of Southern India. Two Volumes, Octavo, November, 1826.

Of all the works which have issued from the medical press of our Asiatic possessions, this is, unquestionably, the most interesting to the Indian practitioner, whether we regard the erudition and research of the author, or the novelty and value of the materials on which he has expended such prodigious labour. Before the publication of the first edition of this work, in 1813, the materia medica of Hindostan was more closely locked up from European eyes, than the most mystic symbols of their mysterious religion. A host of Oriental writers, indeed, among whom the late Sir William Jones shone,

——— Velut inter ignes
Luna minores,———

have unveiled to us the greater part of the complicated Hindoo mythology, (for their system of religion scarcely deserves a better term,) while the manners, customs, castes, arts, agriculture, and domestic economy of the Asiatic nations have been freely portrayed by travellers, merchants, Jesuits, missionaries, and military residents out of number. The diseases, too, by which the European adventurers are scourged in tropical climates, have been investigated with some success, and several scattered notices have been taken of the indigenous diseases of those remote climes; but still we were almost entirely in the dark with respect to the native remedies employed by the Hindoo doctors, as well as the modes of employing them, and the effects thence resulting. The path which Dr. Ainslie pursued was no beaten track, "but winding, and often scarcely to be traced"—overgrown with innumerable noisome as well as useless weeds, "yet occasionally adorned by flowers of rare beauty, and others possessing still more valuable qualities." If he has been so fortunate as to cull a few that may ultimately prove of real utility to mankind, he shall regret neither the time nor the labour that he has bestowed in the search—and may then too be excused for having dragged into public notice some of Nature's fairest offerings, with little to recommend them but—

"A brilliant aspect and an empty name."

The present may almost be considered a new work, so many and so great are the additions. The arrangement and even the title are changed. The work is divided into three distinct parts:—the *first* comprehending such of *our* drugs as are found in India and other Eastern countries, in which division is given some account of their different uses amongst the inhabitants of those regions:—the *second* part containing a description of those medicines which are used almost exclusively by the Hindoos —and the *third*, treating of such articles as are used by them in their arts and manufactures, together with those vegetables which are cultivated as food, embracing a very extensive catalogue of materia alimentaria.

The importance of these volumes to our countrymen in the East, professional, mercantile, and military, will be readily appreciated—and there are few Englishmen, we think, in those regions to whom the labours of our learned and indefatigable author can be indifferent. Under this conviction, we shall dedicate a few pages to the announcement of a work which is rather out of the line of our immediate duty.

On the first part of Dr. Ainslie's work we can have little to say, as it treats of European medicines employed in the East. We shall pass over the whole of the first chapter, and come at once to the second, containing the metals and metallic substances. Even of these, we can advert to but a very few. From Dr. Ainslie's long experience in India, some importance must be attached to his sentiments respecting *mercury*, a medicine so much in use in that quarter of the world. We shall, therefore, stop a few moments to extract a few passages from this portion of the publication.

Cinnabar is found pretty plentifully in some parts of India, and forms a considerable article of commerce. The Hindoos are acquainted with the method of preparing factitious cinnabar, and consider it as *antispasmodic*, and useful in cutaneous complaints, and as a fumigatory in syphilitic ulcers of the nose, mouth, and throat. Calomel and blue-pill are the principal forms of mercury used in India. We shall here introduce a quotation from Dr. Ainslie, on a subject which has lately excited much discussion in this country.

"The use of mercury in venereal complaints, has now been persevered in for upwards of three* hundred years; and although there have of late been doubts entertained with regard to the absolute necessity of it, in such maladies; nay, those who affirm that they can cure the constitutional disease by other and simpler means; I own that I have not been made a convert to this new doctrine, nor shall give up the favorable opinion I have formed of it, after a nearly forty years' experience, notwithstanding all that has been brought forward against it. Much has been said of the *modus operandi* of mercury in syphilis; but perhaps nothing more judicious has been given to the world on that subject, than the following notion of the celebrated Hunter; that the stimulant operation of this metal, induces an action incompatible with the morbid action of the venereal virus, until the poison is either destroyed or evacuated from the body by the excretions; but whatever may be the principle on which it operates, as Dr. Thomson observes, "its efficacy is certain when judiciously† and cautiously administered." It has appeared to me, that after long continued courses of mercury in India, (and they are often, I fear too long,) blood drawn is not only more fluent, but much darker‡ coloured than it appears to be when taken from a person in health; if this position is just, it becomes a question, whether or not this power of liquifying, or partially breaking down the blood, may not extend to the other fluids and secretions of the human body; and so account for, from the use of this medicine, the removal of various glandular and other obstructions, to which the frame is subject, whether buboes, liver affections, tumours of the joints from rheumatism,§ &c. and of this we are certain, that in those painful and, I am sorry to say, frequent hepatic derangements (to be met with in all climates,) and which are particularly distinguished by a dark-coloured,

* "Berengarius Jacobus, a surgeon at Carpo, was the first who cured the venereal disease by means of mercurial ointment; he died in 1527."

† "I know of no failure of a complete cure of syphilis, in India, when the medicine in question was timely resorted to and given with skill, and when the patient lived and managed himself as directed; but I have known infinite mischief produced by delay, carelessness, inattention to diet during the course; these are but too often followed by racking night-pains, nodes, ulcers, and all the rest of the horrid train of anomalous symptoms, which I need not enumerate here."

‡ "I perceive the same power of rendering the blood dark coloured was observed in mercury by Cirillo, a physician of Naples. See Alibert's 'Nouveaux Elémens de Thérapeutique,' vol. ii. p. 268."

§ "Of the wonderful efficacy of mercury in acute rheumatism I have no longer any doubt; it was but lately I saw a delicate female who had been brought to the verge of the grave by bleeding, purging, and the use of diaphoretics, in this complaint, without the smallest advantage, relieved from all her sufferings the moment her mouth became a little affected from the use of the blue pill cautiously administered."

viscid and offensive smelling bile, and a long train of dyspeptic and nervous symptoms, no sooner has the mercury testified its alterative effect on the habit by bringing on a slight soreness of the mouth, than the bile, if examined, will be found to have assumed its proper healthy rhubarb-like appearance and consistence, with that peculiar smell it only has, when secreted by a liver no longer diseased; while the extraventricular digestion will also be observed to go on with its former vigour, and the stomach by sympathy partake of the happy amendment. How the more fluent and darker colour of the blood may be induced by the use of mercury, it is for chemistry to explain; the bright-red colour has been supposed by some to be owing to the iron* it contains; the red globules having been found to consist of a fibrous gluten, and that metal; now we know that mercury forms no amalgam with iron, though it does with gold, copper, zinc, bismuth, and lead, &c.†

"It is well known that the Eastern nations were the first who employed mercury‡ in the cure of some of their obstinate cutaneous and leprous affections; and it may be questioned, whether the natives of India were before the Arabians, or only second in order, in availing themselves of the virtues of that powerful mineral. We are told by Le Clerc, in his "Histoire de la Medecine," (pp. 771, 791,) that, according to Fallopius, the first physicians in Europe who made use of mercury in venereal cases, lived towards the end of the fifteenth century; and that they were induced to make trial of it from what they had read of its efficacy in scabies and leprous cases, in the writings of Rhazes,§ Avicenna,|| and Mesue. The first lived about eight hundred years ago; the second died in 1036; and the last, we are told, published in the twelfth century (though I perceive that Moore, in his History of the Small-pox, says, Mesue lived towards the end of the eighth¶ century, and beginning of the ninth;) and we know that Almenar, a Spaniard, published on it, in 1516.**

---

* "The experiments of the celebrated Rhades show that from twenty-five pounds of blood from the human body, nearly two drachms of the oxide of iron were obtained. See Hooper's Medical Dictionary."

† "See Brande's Manual of Chemistry, vol. ii. p. 260."

‡ "See a valuable paper on the leprosy of the Hindoos, by H. H. Wilson, Esq. in the Transactions of the Medical and Physical Society of Calcutta, vol. i. p. 1."

§ "Argentum vivum cum extinguitur ardens est; quod scabiei auxilium affert." Vide Rhaz. de Re Med. lib, iii. cap. xxiv.

|| "Argentum vivum extinctum adversus pediculos et lendes cum rosaceo oleo valet." Vide Avicen. Canon. Med. lib. ii. tract ii. p. 119.

¶ "But the fact is, there would appear to have been two individuals of the name of Mesue, probably of the same family. The one who has the greatest claim to our notice, is said to have flourished in the tenth century, to have professed the Christian religion, though a native of Bagdat, he practised at Cairo."

** "We learn from Morrison's excellent Chinese Dictionary, that it is not exactly known when mercury was first given, internally, in China; but that in A D. 745, it was termed in that country *puh-sze-che-yo*, or elixir of life."

"Calomel is well known to be most efficacious in liver complaints, in India, and especially in what is called acute hepatitis; but it is not to be prescribed until the more violent inflammatory symptoms have been mitigated by bleeding, blistering, purging, and low diet; it is apt occasionally to open the body too much; in such cases, the admixture of a very small quantity of camphor may be necessary, but certainly not more than a grain, or, at most, two grains in the course of the twenty-four hours. Laudanum or opium in complaints of this nature is often deceitful. I have observed, that calomel is less likely to purge when prescribed in small divided doses during the day, than in a full one at bed time. I commonly gave a grain and a half, or at most two grains, three times in the day, rubbing in ʒi. or ʒij. of mercurial ointment, once in the twenty-four hours, on any part of the body where the skin was soft and free from hair. As soon as the mouth gets properly touched* with the medicine, the pain and uneasiness in the side will be found to abate, so that its further continuance must be regulated with caution. In all cases where offending bile is to be worked off, or where it may be required to excite a new action, calomel is an excellent remedy, mixed, as occasion may require, with other medicines, aloes, jalap, rhubarb, colocynth, &c. It is no place here to enter into a minute investigation respecting the *causes* of diseases; and, no doubt, there has been great difference of opinion concerning those which occasion inflammation of the liver. This much may be safely said, that the stimulus of heat (particularly a dry heat, such, for example, as characterizes the climate of the Coromandel coast, where liver affections are more common than in Bengal or in Malabar,) too full and improper diet, and imprudent potations, have a great share in bringing on the mischief; nor can it be questioned but that a viscid and badly prepared bile, producing obstruction and irritation, is a more immediate source of evil; and so constantly does neglected constipation precede an attack of hepatitis, that we cannot for a moment deny, but that it must powerfully contribute towards hurrying on the organic derangement by binding up what should daily be carried off. How calomel may be supposed to do good under such circumstances, I think may be conceived from what has been above stated regarding the *modus operandi* of mercury on the human frame; viz. by inducing a new action incompatible with the existing evil; but, perhaps, more directly by rendering that bile *more* fluent and natural, which had become viscid and depraved; so the most likely of all things to produce disease by obstruction, stimulated as the liver is at the same time by inordinate heat, and thereby secreting a larger quantity of bile than usual, but which is too thick to flow easily through the various ducts.

"With regard to the proximate causes of hepatitis much has been said by different authors. Winslow ascribed both the acute and chronic to an inflamed state of the ramifications of the vena portæ, which, in his opinion, constitute the seat of the disease. Saunders (and Dr. Good

* "And this is our surest pledge that mercury is to do good."

thinks with some plausibility) suspects the acute variety to be owing to an inflammatory state of the hepatic artery, and the chronic to a like state of the vena portæ, (Study of Medicine, vol. ii. p. 388.)

"When the membranes of the liver are attacked with inflammation, the pain and fever are infinitely more severe than when the substance of that organ is the seat of the disease. Indeed, I have known instances in which it appeared after death, that almost the whole of the parenchyma was converted into pus, though but little pain of any consequence had preceded. The pain stretching up to the top of the right shoulder more especially marks the acute disease; when the inflammation is on the convex surface of the liver the patient lies with most ease on his right side; while, on the other hand, if the concave side is affected, he lies with greater comfort to himself on the left. The acute disease will, invariably, end in suppuration, by which part of the parenchyma of the organ will be destroyed if a stop is not put to the inflammation by bleeding, blistering, and purging, and a subsequent use of mercury. A tumour forming near the edge of the liver, or towards the concave surface, points externally, and can easily be opened, and the patient by this means saved. If the abscess forms on the convex surface, it will point towards the cavity of the thorax, frequently corroding through the diaphragm. I have known several cases in which the inferior lobe of the lungs having contracted an adhesion with those parts of the diaphragm connected with the abscess, the matter was discharged by the bronchiæ; but it more usually happens that the pus is diffused into the cavity of the thorax, and so forms a kind of purulent empyema; when the tumour forms adhesions with the stomach, or colon, the matter is consequently poured into them, and is either vomited up or passed by stool.

"There is a variety of hepatic derangement which is extremely insidious, and is either a sequel of the acute disease, unskillfully treated, or a consequence of general torpor and languid circulation in the organ in question; in the latter instance frequently attacking delicate women of sedentary habits. It is usually accompanied with one or more of the following derangements: obstructions and slight indurations of the liver itself, or in some of the ducts; chronic dysentery; partial feverish symptoms; dyspepsia; great irregularity of the bowels, from vitiated or scantily prepared bile; dejection of spirits; mental irritability; and, if the sufferer be a female, irregularity of the menstrual discharge. This malady requires much nicety in the treatment; mercury must be nsed with infinite caution, cool air sought, and whatever is likely to invigorate the frame without over-stimulating: in such affections calomel, by its cathartic quality, is not so much to be trusted to as the blue pill, an excellent addition to which is ipecacuanha; two or three grains of the blue pill mass, with two grains of ipecacuanha, made into two pills, may be taken in the course of the twenty-four hours." 549.

Dr. Ainslie gives his testimony to the efficacy of mercury in dysentery, and also in those forms of dyspepsia and hypochondriasis which are accompanied, or rather produced by disordered secretion in the liver. The diseases in which mercury has appeared to do harm, in India, are those termed nervous, "whose causes are to be traced rather to the brain than the liver." Where matter is forming or formed, in any part of the body our author considers mercury injurious—indeed he avers that this medicine cannot be made to affect the mouth after suppuration has taken place in the liver. It certainly is extremely difficult to induce ptyalism under such circumstances, but not, we believe, impossible in all cases. In the bilious remittent fevers of hot climates, our author testifies to the utility of mercury—but if, from mismanagement, these fevers assume the typhoid character, then we are not to expect the same effects from this mineral.

Dr. Ainslie next takes up the subject of the *doses* in which calomel ought to be prescribed in diseases of hot climates:—but here our author leaves the safe track of observation, and *assumes* consequences without any previous data. It is evident, from the tenor of Dr. A's remarks that, in his time, or at all events, in his practice, mercury was only given in small doses in Indian diseases. Since that period, a bolder track has been pursued by some Indian practitioners, and this medicine has been given to the amount of 20 grains for a dose in certain diseases of dangerous tendency. Dr. A., without experience in this practice, condemns it unconditionally. As his reasonings are quite unsupported by facts—and, indeed, are no more than gratuitous assumptions, we do not deem it necessary to give them any serious refutation.*

---

* We would beg to draw Dr. Ainslie's attention to the following extracts from a very able paper on fever by Dr. Merrill, in the North American Medical and Surgical Journal, for October, 1826.

"The medical profession has been slow to adopt the plan of administering calomel, which seems so clearly pointed out to them both by reason and experience; and it is only since the masterly works of Dr. James Johnson have been extensively circulated, in America, that the practice of using this medicine in *large doses* has generally obtained. Like many other innovations in medicine, however, this has usually been adopted under such restrictions and modifications, as have rendered it productive of very little advantage, in the management of our most fatal diseases. Physicians, although commonly enthusiastic in their reliance upon newly discovered remedies and principles in medicine, find it exceedingly difficult to divest themselves entirely of long received and established theories. Pride, prejudice, and the authority of books, all combine to produce this effect; and it is a natural consequence, that, however useful or important the innovation may be, when put in common practice, it becomes so amalgamated with preconceived notions, as to impair or wholly destroy its usefulness, and its reputation is lost. This has been the case to a considerable extent with

In the third chapter of the first part, however, Dr. A. has gone again into the subject, in the course of some strictures on Mr. Annesley's late work on Indian diseases; and as he has here stated some practical observations that appear to us extremely well founded, and directly at variance with the opinions so confidently broached by Mr. Annesley in the said work, we shall occupy another page or two with the subject, before we dismiss it.

Our readers will remember that in the 8th No. of this Review, for April, 1826, we gave some account of Mr. Annesley's peculiar opinions respecting the effects of mercury in Indian diseases. This gentleman confidently asserts that the moment the mercury produces an effect on the gums, all its salutary operations are reversed—the energies and "vital resistance" of the system impaired—and, in fact, the hepatitis or dysentery increased instead of being diminished. Let us hear what Dr. Ainslie has to say upon this subject.

" With regard to the modus operandi of mercury, I have already said much under the article Mercury, at p. 540; so shall here briefly observe, that till such time as a certain degree of soreness of the mouth takes place, I have seldom seen any very lasting advantage derived from its use, whether in syphilis, acute rheumatism, remittent fever, hepatitis,* or dysentery; on the contrary, I have but too often found symptoms rendered worse; but the very day, nay, I will go farther, the very hour when the mouth becomes affected, I have invariably remarked, that the disease assumes a more favourable aspect; and this peculiarity is so forcibly impressed on my mind, that I should almost be inclined to

---

the change of practice in regard to the use of calomel. For while it is acknowledged, that the increased magnitude of the dose is an important improvement in the treatment of many diseases, still it is contended that the the usual addition of *opium* must be as necessary as before, and indeed more so, to prevent an immediately cathartic operation—the identical effect required."

" Calomel, when given alone, I have always found to produce the best effects in scruple doses. A smaller quantity than this operates more frequently, producing greater irritation of the stomach and bowels, and causing frequent watery dejections, which rapidly debilitate the patient. In a larger dose, (than 20 grs.) although it does not always create any symptoms immediately unfavourable, yet no additional benefit is observed from it, and it sometimes becomes a source of much irritation, operates too frequently, and produces too great a degree of prostration. While the medium dose of twenty grains rarely fails to quiet the irritability of the stomach and bowels, and carry off large quantities of feculent bilious matter, without griping, tenesmus, prostration, or any other untoward symptoms. This dose may be given more or less frequently, as the nature and violence of the disease may appear to require." 231.

" * See Dr. James Johnson's valuable Medico-Chirurgical Review for April 1826, p. 356."

look upon a slight degree of ptyalism, under the circumstances we alluded to, as a friendly notice from Dame Nature herself, that we had gained the wished-for goal. I shall ever remember one very marked instance, in the case of a poor fellow, a private of his Majesty's 74th regiment, who was in so miserable a state from dysentery, that I expected every hour to be his last; I had quitted him late the preceding evening, when his motions were so frequent, and tenesmus so distressing, that he required the almost constant use of the bed-pan; but what was my surprise in the morning, to find him sitting up in bed; and on being asked how many motions he had during the night, to hear him reply, 'Oh! none since midnight; but I have got a much worse complaint, so sore a mouth, that I can scarcely speak;' he had been for two days rubbing in ℥ij. of strong mercurial ointment, morning and evening, and which fortunately had the effect I so much desired, just in time to save him; the medicine had exerted its *alterative* powers; a transfer of humours had been brought about to a distant part, and the bowels had in consequence been relieved by the change!

"To conclude; when I have spoken of mercury, I meant mercury in all or any of the forms in which it is commonly administered. Calomel, by being a valuable purge, is, no doubt, peculiarly useful in many cases; and, by its at the same time stimulating the liver and biliary ducts, an increased secretion of bile must naturally ensue from its being employed; but I humbly conceive, whatever may have been, certainly very ably, said by Mr. Annesly* respecting the mechanical and chemical operation of calomel, that the happiest influence of that preparation must be by its effect on the general habit, *simply as mercury*; thereby changing for a time the nature of many of the secretions, rendering them evidently more fluent, and, consequently, removing organic congestion, dark viscid bile, &c. And, perhaps, the best of all proofs, in support of what I have here with much diffidence advanced, is our contemplating the extraordinary relief often given in cases of hepatitis, syphilis, acute rheumatism, and, most of all, in dysentery, *when no calomel had been given at all*, but the mercury rubbed in, in the form of an unguent." 651.

With the foregoing extract, which we recommend to the notice of Mr. Annesley, we close the first volume of the work under review.

The second volume opens with some preliminary remarks on the medical writings of the Hindoos. Dr. A. considers it a misfortune that the arts and sciences should ever have been mixed up with the sacred writings of these ancient people. This connexion has been a bar to all improvement. The *Ayur Veda*, or medical writings of antiquity, are considered to be

---

"* See his excellent observations on the use and abuse of calomel, in the first volume (p. 211.) of the Transactions of the Medical Society of Calcutta."

a portion of the fourth Veda, and the work of Brahma himself. This oriental deity instructed a brace of mortals (the sons of the sun) in the art and mystery of physic, and these descendants of Apollo were thenceforth permitted to attend on the gods, when they happened to want a little medicine after a carousal on High Olympus. The *Ayur Veda* originally consisted of one hundred sections, and each section of one thousand stanzas. It was adapted to the limited faculties of man, and consisted of eight divisions:—the first gave instructions for extracting foreign substances, which chance or warfare might have forced into the human body—the second contained ophthalmic and aural surgery (shewing that the oculists and aurists of our days have greater claims to antiquity than they supposed)—the third was general medicine (the business of the general practitioner of our times)—the fourth was the treatment of mental derangement (so that the ancient Hindoos had *mad doctors* as well as the modern Europeans)—the fifth division embraced puerperal and infantile diseases (the Hindoos had their Clarkes, their Merrimans, their Goochs, their Granvilles, their Stones, &c. &c. &c. as well as we have)—the sixth division treated of toxicology (the ancient Orfilas, Magendies, &c.)—seventh division, Chemistry (the Davys, Brandes, &c. of olden time)—but the eighth division is now taken entirely out of the hands of the faculty—the method of *increasing* the population! It is true that many medical writers have generously instructed the world in the mysteries of HYGEIA; but still there seems to be a prevalent idea that the doctor's business is more connected with the decrease than the augmentation of the population.

Both medicine and surgery are now at a very low ebb in India. Dissection is contrary to the tenets of Brahma—and the same doctrine, we are sorry to say, is rapidly spreading among the Brahmins of this country, where anatomy is looked on with horror by the *great unpaid*, in particular. After stating this much respecting their anatomy, it is not of any use to dilate on the surgical acquirements of the Hindoos. The three following extracts from one of their ancient medical books may be considered as curiosities, and containing much truth mixed up with quaint error and superstition.

" ' *Signs of a bilious and irritable habit.* A person of what is called a bilious habit, generally becomes grey very early in life; he is easily made to perspire; his eyes are often inflamed, while his body is pale; he is impatient, perverse, opiniative, and consequential; and for the most part very amorous; the conversation of such an individual is unguarded; he is addicted to falsehood, fond of abstruse studies, yet is he more partial still to the praises that are bestowed on himself.' "

" '*Causes of fever.* An exposure to the heat of the sun, at an early hour of the morning, while fasting; eating voraciously any food of a very hot nature, when the body has been previously weakened by extreme hunger or fatigue; drinking stagnated water, into which withered leaves have fallen; taking a full meal without appetite; unseasonable weather; sudden vicissitudes of temperature: wooded, ill-ventilated valleys; neglected adoration of Crishna; air we have not been accustomed to, whether that of the plains or mountains; the malign influence of an evil spirit or *dewta*; checked perspiration; fear; grief; sleepless nights; long-continued constipation; *in a word, whatever exposes our mortal frame to deviations from its natural and accustomed movements! or clogs nature so much, that it requires great agitation, and consequent heat to bring the body back to sound health.*"

"*What constitutes a good physician.* The sages of antiquity (*maharshies*) have thus handed down to us the qualities which constitute a good physician: he must be a person of strict veracity, and of the greatest sobriety and decorum, holding sexual intercourse with no woman, except his own wife; he ought to be thoroughly skilled in all the commentaries on the *ayurveda*, and be otherwise a man of sense and benevolence; his heart must be charitable; his temper calm; and his constant study how to do good. Such an individual is properly called a good physician, and such a physician ought still daily to improve his mind by an attentive perusal of scientific books (vághádum.)' " xxx.

With this short and imperfect notice of a work which is no more susceptible of analysis than a dictionary, we recommend it to the select library of every European who visits our oriental dominions with the view of residing there for any time.

